# The Under-Appreciated Promiscuity of the Epidermal Growth Factor Receptor Family

**DOI:** 10.3389/fcell.2016.00088

**Published:** 2016-08-22

**Authors:** Sean P. Kennedy, Jordan F. Hastings, Jeremy Z. R. Han, David R. Croucher

**Affiliations:** ^1^Systems Biology Ireland, University College DublinDublin, Ireland; ^2^Kinghorn Cancer Centre, Garvan Institute of Medical ResearchSydney, NSW, Australia; ^3^School of Medicine, University College DublinDublin, Ireland; ^4^St Vincent's Hospital Clinical School, University of New South WalesSydney, NSW, Australia

**Keywords:** EGFR, ERBB2, ERBB3, ERBB4, receptor tyrosine kinase, heterodimerisation, cancer, therapeutic resistance

## Abstract

Each member of the epidermal growth factor receptor (EGFR) family plays a key role in normal development, homeostasis, and a variety of pathophysiological conditions, most notably in cancer. According to the prevailing dogma, these four receptor tyrosine kinases (RTKs; EGFR, ERBB2, ERBB3, and ERBB4) function exclusively through the formation of homodimers and heterodimers within the EGFR family. These combinatorial receptor interactions are known to generate increased interactome diversity and therefore influence signaling output, subcellular localization and function of the heterodimer. This molecular plasticity is also thought to play a role in the development of resistance toward targeted cancer therapies aimed at these known oncogenes. Interestingly, many studies now challenge this dogma and suggest that the potential for EGFR family receptors to interact with more distantly related RTKs is much greater than currently appreciated. Here we discuss how the promiscuity of these oncogenic receptors may lead to the formation of many unexpected receptor pairings and the significant implications for the efficiency of many targeted cancer therapies.

## The epidermal growth factor receptor family

Receptor Tyrosine Kinases (RTKs) are cell surface receptors; all possessing an extracellular ligand binding domain, a single transmembrane domain and an intracellular tyrosine kinase domain (Lemmon and Schlessinger, 2010). RTKs are known to be key regulators of a diverse array of normal cellular functions including cellular metabolism, cell proliferation, migration and differentiation (Schlessinger, 2000). Given this central regulatory role, it is not surprising that mutation or atypical activation of RTKs has emerged as a driver of many diseases, including a variety of different cancers (Yarden and Sliwkowski, 2001; Stern, 2003; Lemmon and Schlessinger, 2010).

There are 58 RTKs which are subdivided into 20 subfamilies (Lemmon and Schlessinger, 2010). One of the most intensely researched RTK families is that of the Epidermal Growth Factor Receptor (EGFR), which consists of four members, the archetypal EGFR, and also ErbB2, ErbB3, and ErbB4. This family of receptors plays an important role in embryonal development of the nervous, gastrointestinal and cardiovascular system (Stern, 2003; Casalini et al., 2004). However, a large body of research has also focused upon their role in cancer, with ectopic expression of EGFR ligands, mutation or overexpression of the receptors apparent in many human cancers (Salomon et al., 1995; Rubin and Yarden, 2001; Yarden and Sliwkowski, 2001; Stern, 2003).

As with many other RTKs, the EGFR family can be activated by the formation of ligand-dependent dimers, both homo-dimers and heterodimers (Lemmon and Schlessinger, 2010). Since the discovery of oligomerization between EGFR family members (Earp et al., 1995; Riese et al., 1995; Riese and Stern, 1998), it has been believed that these hetero-interactions occur exclusively within this family. However, an increasing number of studies now suggest that the EGFR family members are capable of more promiscuous behavior than was originally appreciated, with significant implications for the signaling capacity and therapeutic targeting of these receptors. Therefore, whilst the structure, function and complex dimerisation mechanisms of this receptor family have been the subject of a number of seminal review papers (Rubin and Yarden, 2001; Yarden and Sliwkowski, 2001; Stern, 2003; Hubbard and Miller, 2007; Lemmon, 2009; Lemmon and Schlessinger, 2010), we will focus on the key aspects of the EGFR family necessary to appreciate the possibility and significance of this potential promiscuity.

## EGFR family dimerisation

**Figure 1:**
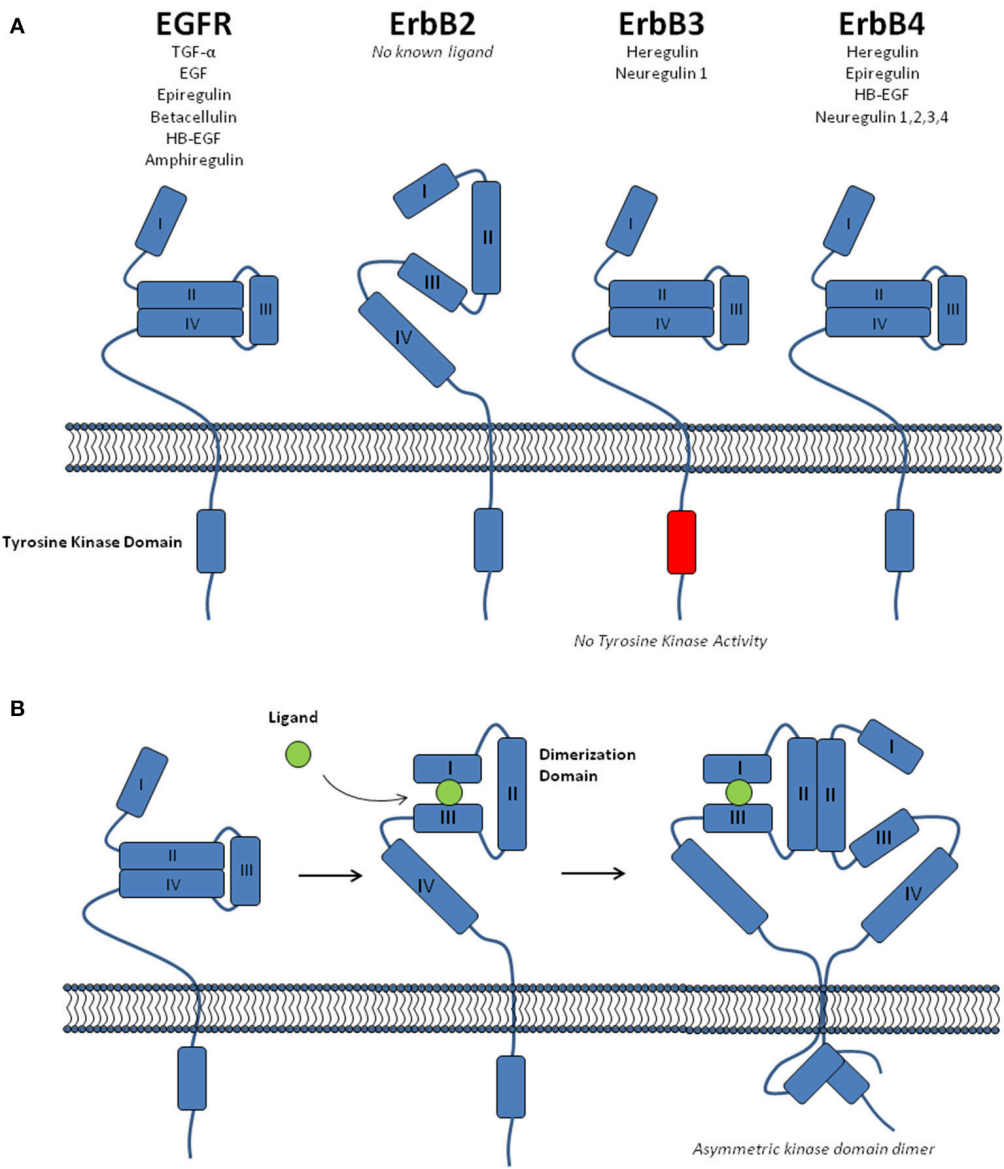
**The EGFR receptor family. (A)** A schematic showing the structure of the four EGFR family receptors (EGFR, ErbB2, ErbB3, and ErbB4), including the four domains within the extracellular region, the intracellular tyrosine kinase domain and the ligands specific to each receptor. EGFR, ErbB3, and ErbB4 are shown in the unliganded, “closed” conformation. **(B)** The conformational change associated with ligand binding, receptor dimerisation and formation of an allosteric kinase domain dimer.

EGFR family members are known to be present at the plasma membrane as both monomers and dimers, including homo- and hetero-dimers (Riese and Stern, 1998; Rubin and Yarden, 2001). The extracellular region of these receptors contains four domains; two β-helix leucine-rich repeat domains that mediate ligand binding (Domains I and III), and two cysteine-rich domains (II and IV) (Figure 1; Lemmon and Schlessinger, 2010). There are 11 different ligands capable of binding the EGFR family members, albeit with differing receptor specificity (Figure 1; Yarden and Sliwkowski, 2001; Lemmon, 2009). This ligand binding is known to promote a conformational change within the receptor, causing an equilibrium shift toward receptor dimerisation (Burgess et al., 2003). The complex mechanisms associated with this process have been the subject of intense research (Hubbard and Miller, 2007; Lemmon, 2009). Although, for the purpose of this review it is sufficient to note the following five key aspects of EGFR family dimerisation; (i) In the absence of ligand EGFR, ErbB3, and ErbB4 adopt a closed conformation that prevents access to a “dimerisation arm” within domain II (Figure 1A). (ii) Upon ligand binding to domains I and III, a conformational change exposes this dimerisation arm, promoting receptor dimerisation (Figure 1B). (iii) There is no known ligand for ErbB2 and it dimerisation arm is constitutively exposed, allowing it to freely form heterodimers with other ligand-bound family members. (iv) Following ligand-mediated receptor dimerisation, the formation of an asymmetric kinase domain dimer promotes allosteric activation of one kinase domain, which then performs *trans*- and auto-phosphorylation of the cytoplasmic tail of both receptors (Zhang et al., 2006; Qiu et al., 2008). (v) The kinase domain of ErbB3 lacks enzyme activity and requires heterodimerisation for *trans*-phosphorylation of its kinase domain (Jura et al., 2009).

There are of course important caveats to this simplified explanation of the normal functioning of EGFR family receptors. At physiological levels of receptor expression, it is believed that ErbB2 is unable to form homo-dimers, and exists either solely as monomers or heterodimers with other ligand bound family members (Yarden and Sliwkowski, 2001). However, it is important to note that upon amplification of the *ERBB2* gene, which occurs frequently in breast cancer (Slamon et al., 1987), the highly elevated levels of ErbB2 lead to violation of these physiological constraints and allow the formation of both ErbB2 homo-dimers and ligand-independent heterodimers (Worthylake et al., 1999; Yarden and Sliwkowski, 2001).

## Signaling mechanisms

Once these receptors have dimerized, *trans*- and auto-phosphorylation of tyrosine residues occurs within the cytoplasmic, C-terminal tail of both receptors (Lemmon and Schlessinger, 2010). These phospho-tyrosine residues lie within amino acid sequence motifs that are recognized by the Src homology 2 (SH2) and phospho-tyrosine binding (PTB) domains of several cytoplasmic and transmembrane adaptor proteins. Each of these receptors in isolation are potentially capable of recruiting a diverse set of interacting proteins through an array of different phosphosites on each receptor (Schulze et al., 2005; Jones et al., 2006). Several well characterized adaptor proteins are known to activate key signaling pathways downstream of EGFR family members, including Shc (Pelicci et al., 1992) and Grb2 (Lowenstein et al., 1992) which bind to all family members and activate MAPK signaling through SOS/Ras, and the PI3K pathway through GAB1 and GAB2 (Yarden and Sliwkowski, 2001; Schulze et al., 2005; Zheng et al., 2013). While EGFR and ErbB2 indirectly activate PI3K in this way, ErbB3, and to a lesser extent, ErbB4 have a key role in activating PI3K signaling through the direct recruitment of the p85 subunit of PI3K (Soltoff et al., 1994; Sepp-Lorenzino et al., 1996; Erlich et al., 2001; Schulze et al., 2005). EGFR and ErbB4 are unique in that they both directly bind and activate STAT5 (Jones et al., 1999; Schulze et al., 2005), while PLCγ can also bind to the activated EGFR (Fedi et al., 1994) and promote PKC activation through the second messenger diacylglycerol.

Due to the distinct adaptor binding profile of each receptor, the formation of heterodimers within the EGFR family has long been thought to diversify the interactome and signaling capacity of these receptors when compared to ligand-dependent receptor homo-dimers (Earp et al., 1995; Riese et al., 1995; Riese and Stern, 1998; Olayioye et al., 2000; Yarden and Sliwkowski, 2001). The signals emanating from these hetero-dimers are known to be significantly stronger than that of ligand-induced homo-dimers (Rubin and Yarden, 2001; Yarden and Sliwkowski, 2001), and this differential signaling output has a significant impact upon the role of each specific dimer in cancer. Experimental studies have shown that the oncogenicity of receptor dimers within the EGFR family is relative to their promiscuity in binding partners (Jones et al., 2006; Kolch and Pitt, 2010), with the ErbB2:ErbB3 heterodimer having the broadest adaptor binding profile and also possessing the most potent transforming capacity (Alimandi et al., 1995; Wallasch et al., 1995; Stern, 2008; Kolch and Pitt, 2010).

In addition to the recruitment of adaptor proteins for signaling pathway activation, various components of the endocytosis machinery also bind to these activated receptor dimers and facilitate internalization of the entire complex (Sorkin and Goh, 2009). This can occur through both clathrin-dependent and independent mechanisms and can result in either signal termination through lysosomal degradation or recycling of the receptors to the plasma membrane (Sorkin and Goh, 2009). This process is also tightly regulated by the distinct binding profile of each receptor, where ErbB2, unlike EGFR, ErbB3, or ErbB4, may have an impaired ability to undergo endocytosis due to a lack of C-terminal internalization signals (Wang et al., 1999; Bertelsen and Stang, 2014). Through this mechanism ErbB2 can potentiate sustained activation and downstream signaling from other EGFR family members through the formation of endocytosis resistant heterodimers or alternative endosomal trafficking (Lenferink et al., 1998; Olayioye et al., 2000; Bertelsen and Stang, 2014).

## The EGFR family in cancer

As receptors to mitogenic growth factors, the EGFR family receptors are heavily involved in the cellular processes governing cell cycle, proliferation, and apoptosis, which makes these receptors important in many human cancers (Stern, 2003; Casalini et al., 2004). In general, the aberrant behavior of RTKs in cancer is characterized by four principal mechanisms: autocrine activation, chromosomal translocations, overexpression, and gain of function mutations (Lemmon and Schlessinger, 2010). The behavior of EGFR family receptors in cancer is predominantly mediated by the latter two of these mechanisms, and overexpression and gain of function mutations of the EGFR family have been extensively documented in the literature.

Both mutation and amplification of the prototype member, EGFR, have been observed in a number of cancers (Normanno et al., 2006; Lemmon and Schlessinger, 2010). Although, these genomic alterations are particularly prevalent in triple-negative breast cancer, with overexpression in over 60% of cases (Simon and FitzGerald, 2016), along with over-expression in 80% of head and neck squamous cell carcinomas (HNSCC) (Alorabi et al., 2016), 60% of non-small-cell lung cancer (Sharma et al., 2007), and 40% of glioblastomas (Westphal et al., 1997). Mutation or overexpression of ErbB2 is regularly observed in lung and gastric cancers, but is more prevalent in breast cancer, where amplification and overexpression of the ErbB2 receptor is strongly linked with aggressiveness of the disease and a poor patient prognosis (Stern, 2008; Creedon et al., 2014). ErbB3 is frequently amplified or over-expressed in breast, lung, liver, colon, gastric, and prostate cancer (Desbois-Mouthon, 2010), and while there is no evidence to suggest that ErbB3 carries oncogenic mutations, somatic mutations have been found to occur in 12% of colorectal cancers (Jaiswal et al., 2013). Although, these mutations do not necessarily confer oncogenic activity upon ErbB3, they have been found to enhance the activation of EGFR upon dimerisation with mutant ErbB3 (Littlefield et al., 2014). The final member of the EGFR family, ErbB4, is not well characterized in cancer and while existing clinical data for the potential role of ErbB4 in breast cancer are contradicting (Gullick, 2003) a number of activating mutations were recently identifed in non-small cell lung cancer (Kurppa et al., 2016).

## Therapeutic targeting

As the receptors of the EGFR family have been identified as potent oncogenes (Stern, 2003), a number of therapeutic agents have been developed in order to interfere and disrupt their roles in cancer cell proliferation and survival. There are two classes of drugs that function in this method: monoclonal antibodies and small molecule tyrosine kinase inhibitors (TKIs). Monoclonal antibodies act on the receptor's extracellular domain, interfering with the receptor through increasing internalization, activating antibody dependent cell-mediated cytotoxicity or disrupting receptor dimerization (Badache and Hynes, 2004). Whereas, TKIs function by inhibiting the cytosolic tyrosine kinase domains through either direct competition with ATP or allosteric inhibition of ATP binding (Levitzki and Mishani, 2006). While both of these therapeutic strategies have had promising results in both the pre-clinical setting as well as in clinical trials, innate and acquired resistance has presented itself as a key challenge for both types of therapies (Nguyen et al., 2009; Oxnard et al., 2011; Stern, 2012; Arteaga and Engelman, 2014; Creedon et al., 2014; D'Amato et al., 2015; Forde and Ettinger, 2015; Landi and Cappuzzo, 2015; Gollamudi et al., 2016; Luque-Cabal et al., 2016).

One prominent example is that of the EGFR targeting monoclonal antibodies cetuximab and panitumumab, which have improved survival for patients with metastatic colorectal cancer harboring a wildtype KRAS (Liu et al., 2016). However, even within this subset of patients, resistance still occurs due to mutations that result in the activation of compensatory signaling pathways (i.e., BRAF, PIK3CA, NRAS, and PTEN; Therkildsen et al., 2014; Liu et al., 2016). Cetuximab is also approved for use in HNSCC, where it is often used in combination with platinum-based chemotherapy and radiation treatment (Sacco and Cohen, 2015; Sacco and Worden, 2016). Despite the high number of HNSCC patients with over-expressed EGFR, there is a distinct lack of biomarkers for response to EGFR-targeting therapies and resistance also occurs through compensatory signaling (Sacco and Cohen, 2015).

Non-small cell lung cancer with EGFR mutations are particularly sensitive to EGFR TKIs erlotinib and gefitinib, however resistance often occurs within 10–14 months of treatment, frequently due to the acquisition of the EGFR T790M “gatekeeper” mutation (Oxnard et al., 2011; Landi and Cappuzzo, 2015). In response to this, next-generation inhibitors are now starting to emerge, including the pan EGFR family inhibitor afatinib, which demonstrates a benefit both as a frontline agent for patients with EGFR mutations and also for those that have relapsed following erlotinib or gefitinib (Hirsh, 2015). However, compensatory signaling through alternative RTKs such as MET or IGF-1R (Nguyen et al., 2009), may represent another method of resistance that might eventually circumvent a pan-EGFR family inhibitor.

ErbB2 overexpression is a highly validated target for monoclonal antibody therapy in breast cancer in both the adjuvant and metastatic setting. The standard of care therapeutic agent is trastuzumab, an antibody that targets ErbB2. However, even when patients are preselected according to ErbB2 expression less than half respond, and most become resistant within a year (Thery et al., 2014). Pertuzumab, an ErbB2 targeting antibody that binds to domain II and inhibits receptor dimerization, has added efficacy and extended survival, especially when used in combination with trastuzumab (Kümler et al., 2014), and is now also approved for use in the neoadjuvant setting (Gollamudi et al., 2016). The dual-targeting TKI Lapatinib, which inhibits both EGFR and ErbB2, is approved for use in ErbB2 positive breast cancer upon relapse following treatment standard chemotherapy and trastuzumab (Nolting et al., 2014). Although, a number of different mechanisms of both acquired and innate resistance to lapatinib have been identified, including compensatory signaling, mutation of ErbB2 and reactivation of the estrogen receptor pathway (D'Amato et al., 2015).

As ErbB3 lacks kinase activity, it cannot be targeted with a small molecule inhibitor. While there are currently no clinically approved monoclonal antibodies that target ErbB3, a number of different strategies are currently being tested (Gaborit et al., 2016). However, in keeping with the established oncogenecity of the ErbB2:ErbB3 heterodimer, recent data from preclinical models also suggests that the simultaneous targeting of ErbB2 and ErbB3 by a bispecific antibody (MM-111) has promising activity as single agent and in combination with trastuzumab (McDonagh et al., 2012). In line with the ability of ErbB3 to form heterodimers with receptors from outside the EGFR family, a bispecific antibody toward the ErbB3:IGF-1R heterodimer has also been developed (Fitzgerald et al., 2014).

Attempts to address this common theme of resistance toward EGFR-family targeted therapies has been met with only partial success (Creedon et al., 2014; D'Amato et al., 2015), indicating that our understanding of acquired resistance in this context is not sufficiently developed. Acquired resistance to EGFR family targeted therapies has been variously attributed to a change in receptor expression or structure, the development of alternative means of activating survival and proliferation pathways governed by the EGFR family, and by a shift in dimerization profile of these receptors (Sergina et al., 2007; Stern, 2012; Arteaga and Engelman, 2014; Creedon et al., 2014; Luque-Cabal et al., 2016). While a shift in dimerization profile is commonly accepted as a mechanism conferring resistance to EGFR family targeted therapies, this avenue of thought has not been completely explored. Historically, it has been accepted that EGFR family receptors only form dimers with other members of the family. However, studies have now emerged indicating that due to the plasticity of these receptors, the pool of potential heterodimers is much larger than previously thought. Therefore, a thorough understanding of these potential interaction partners and their effect on EGFR family signaling pathways is needed to provide specific cancer therapies that overcome the challenge of acquired resistance.

## Alternative hetero-interactions for EGFR family members

In the two decades since the discovery of heterodimerisation between EGFR family members (Earp et al., 1995; Riese et al., 1995; Riese and Stern, 1998), the dogma in this field has kept these interactions strictly within the family. Whilst this restricted view may indeed hold at physiological levels of receptor expression, the amplification and up-regulation of each of these receptors in cancerous tissue suggest that within patho-physiological settings the capability of these receptors to form heterodimers with each other, and indeed other more distantly related RTKs, is greatly increased. This is particularly likely in the case of breast cancer cells where ErbB2 is amplified and over-expressed, leading to the formation of ErbB2-containing heterodimers in a ligand-independent manner (Worthylake et al., 1999; Yarden and Sliwkowski, 2001).

This hypothesis of EGFR family promiscuity is supported by many experimental observations of RTKs from outside of the EGFR family interacting with either EGFR, ErbB2, or ErbB3 (Table 1). Although, no alternative RTK interactions with ErbB4 could be found in the literature. Of these alternative RTKs, MET, IGF-1R and AXL were all observed to interact with EGFR, ErbB2, and ErbB3. Whilst RET and FGFR2 also interacted with both EGFR and ErbB3. Interestingly, in these studies MET was also observed interacting with RET (Tanizaki et al., 2011; Meyer et al., 2013), AXL (Meyer et al., 2013), and FGFR2 (Chang et al., 2015), while AXL was observed interacting with PDGFRb (Meyer et al., 2013), suggesting that the potential combinatorial hetero-interactions between RTKs may be even more complex than we are currently proposing. With the exception of two studies, all of these observed hetero-interactions occurred within cancer cell lines that were either amplified or over-expressing one of the interacting receptors. These findings suggest that while specific heterodimers, such as EGFR:IFG-1R, may have important physiological roles (Ahmad et al., 2004), the over-expression or amplification of RTKs within this patho-physiological setting may be driving the formation of complex receptor interactions that would not otherwise occur in normal biology.

**Table 1:** **Alternative receptor tyrosine kinases observed to form heterodimers with members of the EGFR family**.

**EGFR family member**	**Interacting RTK**	**Detection method**	**Sufficient controls**	**Sufficient loading**	**Functional outcome of heterodimer formation**	**Tissue**	**References**
EGFR	FGFR2	co-IP	No	No	Resistance to FGFR2 inhibitor (AZD4547), possibly through ERK activation.	SNU16 and KATOIII gastric cancer cell lines	Chang et al., 2015
	AXL	Cross-linking co-IP	Yes	Yes	Ligand induced, EGFR mediated phosphorylation of AXL. Increased Akt signaling, cell motility and resistance to erlotinib.	MDA-MB-231 and MCF-7 breast cancer cell lines	Meyer et al., 2013
		MS	Yes	Yes	HGF-induced EGFR inhibition, resulting in increased interaction and activation of AXL.	SCC9 HNSCC cell line	Gusenbauer et al., 2013
		co-IP	No	Yes			
	EPHA2	MS	Yes	Yes	HGF-induced EGFR inhibition, resulting in increased interaction and activation of EphA2.	SCC9 HNSCC cell line	Gusenbauer et al., 2013
		co-IP	No	Yes			
	IGF1R	co-IP	Yes	No	Heterodimer induced by both IGF-1 and EGF. Increased IGF-1 induced ERK activation.	Normal mammary epithelial cells	Ahmad et al., 2004
	MET	MS	Yes	Yes	HGF-induced EGFR inhibition through ERK signaling. Resistance to gefitinib.	SCC9 HNSCC cell line	Gusenbauer et al., 2013
		co-IP	Yes	Yes	Transactivation of EGFR by MET in MET amplified cells. Depletion of EGFR inhibited ERK and AKT activation, promoting apoptosis.	EBC-1 and H1993 non-small lung cancer cell lines	Tanizaki et al., 2011
		co-IP	No	Yes	EGF or TGFα induced unidirectional transactivation of MET by EGFR.	HepG2, AKN-1, and HuH6 human hepatoma cell lines. A431 human epidermoid carcinoma cell line	Jo et al., 2000
		co-IP	Yes	Yes	c-Src dependent transactivation of MET by EGFR, in the absence of HGF. Promotes cell proliferation and resistance to EGFR inhibitors.	SUM229 breast cancer cell line	Mueller et al., 2010
	PDGFR	co-IP	No	Yes	Heterodimers detected, no function attributed.	Malignant peripheral nerve sheath tumor samples	Perrone et al., 2009
		Cross-linking co-IP	Yes	Yes	c-Src dependent transactivation of EGFR by PDGFRβ, following PDGF stimulation. Promotes ERK activation.	Rat aortic vascular smooth muscle cells	Saito et al., 2001
		co-IP	Yes	No	Ligand independent transactivation of PDGFRβ by EGFR observed in EGFR over-expressing cells.	COS-7 and Hs27 cell lines	Habib et al., 1998
	RET	co-IP	Yes	Yes	EGF dependent transactivation of RET by EGFR. Promotion of cell proliferation.	PCCL3 papillary thyroid carcinoma cell line	Croyle et al., 2008
ErbB2	MET	co-IP	Yes	Yes	Transactivation of ErbB2 by MET in MET amplified cells. Depletion of ErbB2 inhibited ERK and AKT activation, promoting apoptosis, and STAT3 activation, inhibiting migration.	EBC-1 and H1993 non-small lung cancer cell lines	Tanizaki et al., 2011
	AXL	Cross-linking co-IP	Yes	Yes	Inferred resistance to lapatinib.	MDA-MB-231 and MCF-7 breast cancer cell lines	Meyer et al., 2013
	NTRK1	co-IP	Yes	Yes	NGF induced transactivation of ErbB2 by NTRK1, promoting ERK activation and proliferation.	SKBR3 breast cancer cell line	Tagliabue et al., 2000
	IGF-1R	co-IP	Yes	Yes	Transactivation of ErbB2 by IGF-1R. Interaction induced by heregulin and IGF-1.	C4HD and MCF-7 breast cancer cell lines	Balañá et al., 2001
		co-IP	No	Yes	Transactivation of ErbB2 by IGF-1R, leading to trastuzumab resistance.	SKBR3 breast cancer cell line	Nahta et al., 2005
		co-IP	No	Yes	Hetero-dimer present in trastuzumab resistant cells. Proposed hetero-trimer with ErbB3.	SKBR3 and BT474 breast cancer cell line	Huang et al., 2010
		co-IP	Yes	Yes	Dual targeting of ErbB2:IGF-1R heterodimer increases sensitivity to trastuzumab.	SKBR3 and BT474 breast cancer cell line	Browne et al., 2011
ErbB3	RET	PLA, co-IP (transfected ErbB3)	Yes	Yes	May promote vandetanib resistance.	1765–92 Myxoid cell line	Safavi et al., 2016
	AXL	Cross-linking co-IP	Yes	Yes	Inferred resistance to ErbB3 targeted therapy.	MDA-MB-231 and MCF-7 breast cancer cell lines	Meyer et al., 2013
	MET	co-IP	Yes	Yes	Transactivation of ErbB3 by MET, in MET amplified cells. Depletion of ErbB3 inhibited ERK and AKT activation, promoting apoptosis.	EBC-1 and H1993 non-small lung cancer cell lines	Tanizaki et al., 2011
		co-IP	No	Yes	Transactivation of ErbB3 by MET in MET amplified cells. Promotes PI3K pathway activation and resistance to gefitinib	HCC827 NSCLC cell line	Engelman et al., 2007
	IGF-1R	co-IP	No	Yes	Promotes trastuzumab resistance, proposed hetero-trimer with ErbB2.	SKBR3 and BT474 breast cancer cell line	Huang et al., 2010
	FGFR2	co-IP	No	No	Resistance to FGFR2 inhibitor (AZD4547), possibly through ERK activation.	SNU16 and KATOIII gastric cancer cell lines	Chang et al., 2015

With only one exception, co-immunoprecipitation was the only experimental technique utilized to observe all of these receptor interactions (Table 1). Additionally, a number of these studies did not utilize sufficient non-specific binding controls or include satisfactory data to demonstrate enrichment of the target RTK or confirm equal loading. This lack of controls is especially evident where the interpretation of immunoprecipitation results has been confounded by the presence of antibody based targeted therapies. This particular problem was previously observed for the ErbB2:IGF-1R heterodimer, where the dimer was strongly detected following IGF-1R immunoprecipitation in Trastuzumab resistant SKBR3 and BT474 cells that were cultured continuously in the presence of Trastuzumab (Nahta et al., 2005; Huang et al., 2010). Due to a lack of sufficient non-specific binding controls, this strong interaction was later confirmed to be mediated instead by direct immunoprecipitation of ErbB2 by Trastuzumab (Browne et al., 2011), although this heterodimer was still detected in the absence of Trastuzumab, albeit at a somewhat lower level (Browne et al., 2011). Despite this, the number of adequately controlled experiments identifying these non-canonical interactions suggests that it is now important that more advanced techniques are applied to the investigation of these potential heterodimers.

## Structural considerations

The structural nature of these interactions also needs to be established, as co-immunoprecipitation data does not confirm a direct, physical interaction between these two receptors, and could also be interpreted that they are contained merely within the same multi-molecular complex. The interaction of EGFR family receptors with a wider array of RTKs is also potentially complicated by the differing dimerisation mechanisms present within the different families of RTKs (Lemmon and Schlessinger, 2010). Dimerisation within the EGFR family occurs entirely through contacts between the two receptor molecules (Garrett et al., 2002; Ogiso et al., 2002), whereas NTRK1 dimerisation is entirely ligand-mediated (Wehrman et al., 2007) and FGFR family dimers form through a combination of both ligand and receptor-mediated contacts, with the addition of heparin-mediated stabilization (Schlessinger et al., 2000). However, despite their mechanistic differences, both NTRK1 and FGFR2 have been observed interacting with EGFR family receptors (Table 1). It is therefore tempting to speculate that the formation of heterodimers between RTKs with vastly different mechanisms of ligand binding and extracellular domain interaction, could instead be mediated through interactions at the transmembrane or intracellular region. In support of this, the transmembrane alpha helices of EGFR family receptors can also mediate both ligand-dependent and -independent interactions (Burke and Stern, 1998; Mendrola et al., 2002; Gerber et al., 2004). GXXXG motifs within the transmembrane regions are considered to be a general dimerisation motif for transmembrane helices (Lemmon et al., 1994), which promote hetero-interactions within the EGFR family (Escher et al., 2009) but are notably present within a number of different RTKs (Mendrola et al., 2002). These GXXXG motifs are thought to maintain the correct alignment of kinase domains within inactive, pre-formed receptor dimers and also facilitate formation of the activated assymetrical kinase dimer upon ligand binding (Kovacs et al., 2015). However, these motifs may also facilitate a wider range of receptor interactions (Sawma et al., 2014). From the studies identified in Table 1, it is also notable that the EGFR-RET association did not require the extracellular domain of RET (Croyle et al., 2008), confirming that these interactions can occur solely through transmembrane or cytosolic contacts.

The role of ligand binding also needs to be investigated within this expanded paradigm. From the studies that observed non-canonical RTK interactions (Table 1), one study using normal mammary epithelial cells noticed an increase in EGFR:IGF-1R dimerisation following either EGF or IGF-1 treatment (Ahmad et al., 2004). In contrast, many other studies using cancer cell lines noted that various heterodimers were already present under basal conditions and that ligand stimulation of one receptor increased *trans*-phosphorylation of its interacting partner (Habib et al., 1998; Jo et al., 2000; Tagliabue et al., 2000; Saito et al., 2001; Croyle et al., 2008; Meyer et al., 2013). One of these studies also noted that EGFR:MET heterodimers were detectable in a ligand-independent manner in hepatoma cells, but absent in normal hepatocytes, even in the presence of ligand (Jo et al., 2000). However, another detected EGFR:PDGFRβ heterodimers in rat aortic vascular smooth muscle cells in the absence of ligand (Saito et al., 2001). These potentially conflicting findings may be reconciled when it is considered that Src activity was often required for the formation of these heterodimers (Saito et al., 2001; Mueller et al., 2010), suggesting that while elevated receptor expression may play a significant role in the formation of ligand-independent hetero-dimers, the signaling state of the cell is also an important consideration. Nonetheless, the bulk of these studies suggest that cancerous cells may present a uniquely rich environment of non-physiological receptor interactions that greatly alter the signaling landscape of these cells, but may also render them amenable to therapeutic targeting.

## Therapeutic implications

One common functional outcome associated with the formation of these alternative hetero-dimers is the emergence of resistance to targeted therapy toward either interacting receptor (Table 1). A potential mechanism for this therapeutic resistance is the ability of EGFR family members to co-opt signaling capacity through these alternative receptor interactions. Due to an increase in interactome diversity, the signaling capacity of EGFR family heterodimers is significantly stronger than that of homodimers (Rubin and Yarden, 2001), and the oncogenicity of these receptor dimers is also relative to their interactome diversity (Jones et al., 2006). Through the formation of an expanded repertoire of heterodimers with more distantly related RTKs, there is an even greater potential for increased interactome diversity for these alternative heterodimers. There is some emerging recognition of this potential resistance mechanism in the development of bivalent antibody therapy targeting the ErbB3:IGF-1R heterodimer (Fitzgerald et al., 2014). In this study, the concurrent inhibition of both IGF-1R and ErbB3 prevented the emergence of compensatory PI3K/mTOR signaling associated with IGF-1R TKI treatment. Many of the studies in Table 1 also implicated signaling pathways activated by aberrant heterodimers in the resistance to targeted therapy. This most commonly involved ERK (Tagliabue et al., 2000; Saito et al., 2001; Ahmad et al., 2004; Tanizaki et al., 2011; Chang et al., 2015) or Akt (Tanizaki et al., 2011) activation, and suggests that rationalized therapeutic strategies capable of specifically targeting these heterodimers may prevent resistance occurring via downstream pathway compensation.

The role of targeted therapeutics in the inhibition/formation of these alternative heterodimers also needs to be addressed. Interestingly, previous observations have noted an increased *trans*-phosphorylation of EphA2 by ErbB2 following treatment with Trastuzumab (Zhuang et al., 2010). Although the potential for heterodimerisation between these two RTKs was not addressed in this study, a number of other studies have demonstrated the ability of alternative RTKs to mediate resistance to ErbB2 monoclonal antibodies (Table 1). Given that these monoclonal antibodies target the extracellular regions of these receptors, and intracellular/transmembrane regions may be involved in promoting heterodimerisation with alternative RTKs, the possibility that these artificial ligands may promote a shift in the dimerisation profile of their target receptor should be thoroughly addressed.

The formation of alternative heterodimers may also impact the efficacy of TKIs targeting EGFR or ErbB2. These small molecule inhibitors have been shown to increase the interaction between heterodimers within the EGFR family (Macdonald-Obermann et al., 2013) and also lead to sub-cellular redistribution and compensation by alternative receptors, including ErbB3 (Sergina et al., 2007). From a mechanistic viewpoint, the ability of these TKIs to inhibit the kinase activity of EGFR and/or ErbB2 may not prevent the kinase domain of that receptor acting as an allosteric activator for the kinase domain of an alternative interaction partner, and thereby undergoing *trans*-phosphorylation despite the presence of a kinase inhibitor. This was noted in studies of MET amplified lung cancer cells, which demonstrated that the formation of EGFR:MET (Mueller et al., 2010; Tanizaki et al., 2011) and ErbB2:MET (Tanizaki et al., 2011) heterodimers rendered the EGFR family member resistant to dephosphorylation in the presence of their respective TKI (Tanizaki et al., 2011). A similar observation was also made for the EGFR:PDGFRβ (Saito et al., 2001) and ErbB2:NTRK1 (Tagliabue et al., 2000) heterodimers, in which EGFR family member auto-phosphorylation occurred following ligand activation of the interacting receptor, even in the presence of their respective kinase domain inhibitor. A similar mechanism was also observed for mutationally inactivated kinase domain (Deb et al., 2001), where an EGF-bound mutant EGFR could still induce MAPK and PI3K pathway activation through ErbB2. Taken together, all of these observations suggest that the formation of alternative heterodimers may still serve to increase the interactome diversity and signaling capacity of EGFR family containing heterodimers, even in the presence of TKIs.

## Future perspectives

With the exception of two studies (Gusenbauer et al., 2013; Meyer et al., 2013), there has been little effort to systematically investigate the wide array of potential heterodimers that may form with EGFR family members. Given the wide-ranging nature of these oncogenic receptors, their frequent therapeutic targeting and proclivity for adaptation and therapeutic resistance, focus should be placed upon their ability to distribute their oncogenic signaling capacity across an array of potential RTK interacting partners. The increased interactome diversity and signaling potential associated with the formation of these alternative heterodimers is also an important avenue of research. In this regard, we recently developed a novel affinity purification technique that facilitates the specific isolation and proteomic characterization of receptor dimers, and indeed any other protein dimer of interest (Croucher et al., 2016). We applied this technique to investigate the interactome of ErbB2 in the form of a homodimer or a heterodimer with either EGFR or ErbB3. This analysis revealed dimer-specific interaction patterns for key adaptor proteins and also identified a number of novel interacting partners that underlie the signaling capacity of each receptor dimer. In the future, the application of such techniques to alternative heterodimers will facilitate the detailed investigation of their interactomes and signaling capacity, potentially identifying new therapeutic strategies to target these heterodimers.

As it becomes increasingly apparent that monotherapy is an inefficient strategy for therapeutically targeting signaling molecules in cancer cells, more elegant approaches will need to be developed that encapsulate the plasticity and adaptability of their extant network structure. The promiscuous EGFR receptor family members and their many interaction partners sit at the top of many of these network structures, and their surface availability will continue to make them attractive therapeutic targets. Hopefully, as we begin to unravel their under-appreciated mechanisms for generating therapeutic resistance we can move toward effective and durable strategies to target these potent oncogenes.

## Author contributions

SK, JFH, and JZRH reviewed the literature and wrote the manuscript. DC supervised SK, JFH, and JZRH, conceived the idea, reviewed the literature, wrote and edited the manuscript.

### Conflict of interest statement

The authors declare that the research was conducted in the absence of any commercial or financial relationships that could be construed as a potential conflict of interest.

## References

[B1] Ahmad T. Farnie G. Bundred N. J. Anderson N. G. (2004). The mitogenic action of insulin-like growth factor I in normal human mammary epithelial cells requires the epidermal growth factor receptor tyrosine kinase. J. Biol. Chem. 279, 1713–1719. 10.1074/jbc.M306156200 14593113

[B2] Alimandi M. Romano A. Curia M. C. Muraro R. Fedi P. Aaronson S. A.. (1995). Cooperative signaling of ErbB3 and ErbB2 in neoplastic transformation and human mammary carcinomas. Oncogene 10, 1813–1821. 7538656

[B3] Alorabi M. Shonka N. A. Ganti A. K. (2016). EGFR monoclonal antibodies in locally advanced head and neck squamous cell carcinoma: what is their current role? Crit. Rev. Oncol. Hematol. 99, 170–179. 10.1016/j.critrevonc.2015.12.006 26797287

[B4] Arteaga C. L. Engelman J. A. (2014). ERBB receptors: from oncogene discovery to basic science to mechanism-based cancer therapeutics. Cancer Cell 25, 282–303. 10.1016/j.ccr.2014.02.025 24651011 PMC4018830

[B5] Badache A. Hynes N. E. (2004). A new therapeutic antibody masks ErbB2 to its partners. Cancer Cell 5, 299–301. 10.1016/S1535-6108(04)00088-1 15093533

[B6] Balañá M. E. Labriola L. Salatino M. Movsichoff F. Peters G. Charreau E. H.. (2001). Activation of ErbB-2 via a hierarchical interaction between ErbB-2 and type I insulin-like growth factor receptor in mammary tumor cells. Oncogene 20, 34–47. 10.1038/sj.onc.1204050 11244498

[B7] Bertelsen V. Stang E. (2014). The mysterious ways of ErbB2/HER2 trafficking. Membranes (Basel). 4, 424–446. 10.3390/membranes4030424 25102001 PMC4194043

[B8] Browne B. C. Crown J. Venkatesan N. Duffy M. J. Clynes M. Slamon D.. (2011). Inhibition of IGF1R activity enhances response to trastuzumab in HER-2-positive breast cancer cells. Ann. Oncol. 22, 68–73. 10.1093/annonc/mdq349 20647220

[B9] Burgess A. W. Cho H. S. Eigenbrot C. Ferguson K. M. Garrett T. P. Leahy D. J.. (2003). An open-and-shut case? Recent insights into the activation of EGF/ErbB receptors. Mol. Cell 12, 541–552. 10.1016/S1097-2765(03)00350-2 14527402

[B10] Burke C. L. Stern D. F. (1998). Activation of Neu (ErbB-2) mediated by disulfide bond-induced dimerization reveals a receptor tyrosine kinase dimer interface. Mol. Cell. Biol. 18, 5371–5379. 10.1128/MCB.18.9.5371 9710621 PMC109122

[B11] Casalini P. Iorio M. V. Galmozzi E. Ménard S. (2004). Role of HER receptors family in development and differentiation. J. Cell. Physiol. 200, 343–350. 10.1002/jcp.20007 15254961

[B12] Chang J. Wang S. Zhang Z. Liu X. Wu Z. Geng R.. (2015). Multiple receptor tyrosine kinase activation attenuates therapeutic efficacy of the fibroblast growth factor receptor 2 inhibitor AZD4547 in FGFR2 amplified gastric cancer. Oncotarget 6, 2009–2022. 10.18632/oncotarget.2987 25576915 PMC4385832

[B13] Creedon H. Byron A. Main J. Hayward L. Klinowska T. Brunton V. G. (2014). Exploring mechanisms of acquired resistance to HER2 (human epidermal growth factor receptor 2)-targeted therapies in breast cancer. Biochem. Soc. Trans. 42, 822–830. 10.1042/BST20140109 25109964

[B14] Croucher D. R. Iconomou M. Hastings J. F. Kennedy S. P. Han J. Z. R. Shearer R. F.. (2016). Bimolecular complementation affinity purification (BiCAP) reveals dimer-specific protein interactions for ERBB2 dimers. Sci. Signal. 9:ra69. 10.1126/scisignal.aaf0793 27405979

[B15] Croyle M. Akeno N. Knauf J. A. Fabbro D. Chen X. Baumgartner J. E.. (2008). RET/PTC-induced cell growth is mediated in part by epidermal growth factor receptor (EGFR) activation: evidence for molecular and functional interactions between RET and EGFR. Cancer Res. 68, 4183–4191. 10.1158/0008-5472.CAN-08-0413 18519677 PMC4341915

[B16] D'Amato V. Raimondo L. Formisano L. Giuliano M. De Placido S. Rosa R.. (2015). Mechanisms of lapatinib resistance in HER2-driven breast cancer. Cancer Treat. Rev. 41, 877–883. 10.1016/j.ctrv.2015.08.001 26276735

[B17] Deb T. B. Su L. Wong L. Bonvini E. Wells A. David M.. (2001). Epidermal growth factor (EGF) receptor kinase-independent signaling by EGF. J. Biol. Chem. 276, 15554–15560. 10.1074/jbc.M100928200 11279155

[B18] Desbois-Mouthon C. (2010). The HER3/ErbB3 receptor: a promising target in cancer drug therapy. Gastroenterol. Clin. Biol. 34, 255–259. 10.1016/j.gcb.2010.03.002 20418034

[B19] Earp H. S. Dawson T. L. Li X. Yu H. (1995). Heterodimerization and functional interaction between EGF receptor family members: a new signaling paradigm with implications for breast cancer research. Breast Cancer Res. Treat. 35, 115–132. 10.1007/BF00694752 7612898

[B20] Engelman J. A. Zejnullahu K. Mitsudomi T. Song Y. Hyland C. Park J. O.. (2007). MET amplification leads to gefitinib resistance in lung cancer by activating ERBB3 signaling. Science 316, 1039–1043. 10.1126/science.1141478 17463250

[B21] Erlich S. Goldshmit Y. Lupowitz Z. Pinkas-Kramarski R. (2001). ErbB-4 activation inhibits apoptosis in PC12 cells. Neuroscience 107, 353–362. 10.1016/S0306-4522(01)00350-5 11731109

[B22] Escher C. Cymer F. Schneider D. (2009). Two GxxxG-like motifs facilitate promiscuous interactions of the human ErbB transmembrane domains. J. Mol. Biol. 389, 10–16. 10.1016/j.jmb.2009.04.002 19361517

[B23] Fedi P. Pierce J. H. di Fiore P. P. Kraus M. H. (1994). Efficient coupling with phosphatidylinositol 3-kinase, but not phospholipase C gamma or GTPase-activating protein, distinguishes ErbB-3 signaling from that of other ErbB/EGFR family members. Mol. Cell. Biol. 14, 492–500. 10.1128/MCB.14.1.492 8264617 PMC358399

[B24] Fitzgerald J. B. Johnson B. W. Baum J. Adams S. Iadevaia S. Tang J.. (2014). MM-141, an IGF-IR- and ErbB3-directed bispecific antibody, overcomes network adaptations that limit activity of IGF-IR inhibitors. Mol. Cancer Ther. 13, 410–425. 10.1158/1535-7163.MCT-13-0255 24282274

[B25] Forde P. M. Ettinger D. S. (2015). Managing acquired resistance in EGFR-mutated non-small cell lung cancer. Clin. Adv. Hematol. Oncol. 13, 528–532. 26351816

[B26] Gaborit N. Lindzen M. Yarden Y. (2016). Emerging anti-cancer antibodies and combination therapies targeting HER3/ERBB3. Hum. Vaccin. Immunother. 12, 576–592. 10.1080/21645515.2015.1102809 26529100 PMC4964743

[B27] Garrett T. P. McKern N. M. Lou M. Elleman T. C. Adams T. E. Lovrecz G. O.. (2002). Crystal structure of a truncated epidermal growth factor receptor extracellular domain bound to transforming growth factor alpha. Cell 110, 763–773. 10.1016/S0092-8674(02)00940-6 12297049

[B28] Gerber D. Sal-Man N. Shai Y. (2004). Two motifs within a transmembrane domain, one for homodimerization and the other for heterodimerization. J. Biol. Chem. 279, 21177–21182. 10.1074/jbc.M400847200 14985340

[B29] Gollamudi J. Parvani J. G. Schiemann W. P. Vinayak S. (2016). Neoadjuvant therapy for early-stage breast cancer: the clinical utility of pertuzumab. Cancer Manag. Res. 8, 21–31. 10.2147/CMAR.S55279 26937204 PMC4762586

[B30] Gullick W. J. (2003). c-erbB-4/HER4: friend or foe? J. Pathol. 200, 279–281. 10.1002/path.1335 12845622

[B31] Gusenbauer S. Vlaicu P. Ullrich A. (2013). HGF induces novel EGFR functions involved in resistance formation to tyrosine kinase inhibitors. Oncogene 32, 3846–3856. 10.1038/onc.2012.396 23045285

[B32] Habib A. A. Högnason T. Ren J. Stefánsson K. Ratan R. R. (1998). The epidermal growth factor receptor associates with and recruits phosphatidylinositol 3-kinase to the platelet-derived growth factor beta receptor. J. Biol. Chem. 273, 6885–6891. 10.1074/jbc.273.12.6885 9506992

[B33] Hirsh V. (2015). Next-generation covalent irreversible kinase inhibitors in NSCLC: focus on Afatinib. BioDrugs 29, 167–183. 10.1007/s40259-015-0130-9 26123538 PMC4488453

[B34] Huang X. Gao L. Wang S. McManaman J. L. Thor A. D. Yang X.. (2010). Heterotrimerization of the growth factor receptors erbB2, erbB3, and insulin-like growth factor-i receptor in breast cancer cells resistant to herceptin. Cancer Res. 70, 1204–1214. 10.1158/0008-5472.CAN-09-3321 20103628

[B35] Hubbard S. R. Miller W. T. (2007). Receptor tyrosine kinases: mechanisms of activation and signaling. Curr. Opin. Cell Biol. 19, 117–123. 10.1016/j.ceb.2007.02.010 17306972 PMC2536775

[B36] Jaiswal B. S. Kljavin N. M. Stawiski E. W. Chan E. Parikh C. Durinck S.. (2013). Oncogenic ERBB3 mutations in human cancers. Cancer Cell 23, 603–617. 10.1016/j.ccr.2013.04.012 23680147

[B37] Jo M. Stolz D. B. Esplen J. E. Dorko K. Michalopoulos G. K. Strom S. C. (2000). Cross-talk between epidermal growth factor receptor and c-Met signal pathways in transformed cells. J. Biol. Chem. 275, 8806–8811. 10.1074/jbc.275.12.8806 10722725

[B38] Jones F. E. Welte T. Fu X. Y. Stern D. F. (1999). ErbB4 signaling in the mammary gland is required for lobuloalveolar development and Stat5 activation during lactation. J. Cell Biol. 147, 77–88. 10.1083/jcb.147.1.77 10508857 PMC2164978

[B39] Jones R. B. Gordus A. Krall J. A. MacBeath G. (2006). A quantitative protein interaction network for the ErbB receptors using protein microarrays. Nature 439, 168–174. 10.1038/nature04177 16273093

[B40] Jura N. Shan Y. Cao X. Shaw D. E. Kuriyan J. (2009). Structural analysis of the catalytically inactive kinase domain of the human EGF receptor 3. Proc. Natl. Acad. Sci. U.S.A. 106, 21608–21613. 10.1073/pnas.0912101106 20007378 PMC2791034

[B41] Kolch W. Pitt A. (2010). Functional proteomics to dissect tyrosine kinase signalling pathways in cancer. Nat. Rev. Cancer 10, 618–629. 10.1038/nrc2900 20720570

[B42] Kovacs E. Zorn J. A. Huang Y. Barros T. Kuriyan J. (2015). A structural perspective on the regulation of the epidermal growth factor receptor. Annu. Rev. Biochem. 84, 739–764. 10.1146/annurev-biochem-060614-034402 25621509 PMC4452390

[B43] Kümler I. Tuxen M. K. Nielsen D. L. (2014). A systematic review of dual targeting in HER2-positive breast cancer. Cancer Treat. Rev. 40, 259–270. 10.1016/j.ctrv.2013.09.002 24080156

[B44] Kurppa K. J. Denessiouk K. Johnson M. S. Elenius K. (2016). Activating ERBB4 mutations in non-small cell lung cancer. Oncogene 35, 1283–1291. 10.1038/onc.2015.185 26050618

[B45] Landi L. Cappuzzo F. (2015). Experience with erlotinib in the treatment of non-small cell lung cancer. Ther. Adv. Respir. Dis. 9, 146–163. 10.1177/1753465815588053 26063687

[B46] Lemmon M. A. (2009). Ligand-induced ErbB receptor dimerization. Exp. Cell Res. 315, 638–648. 10.1016/j.yexcr.2008.10.024 19038249 PMC2667204

[B47] Lemmon M. A. Schlessinger J. (2010). Cell signaling by receptor tyrosine kinases. Cell 141, 1117–1134. 10.1016/j.cell.2010.06.011 20602996 PMC2914105

[B48] Lemmon M. A. Treutlein H. R. Adams P. D. Brünger A. T. Engelman D. M. (1994). A dimerization motif for transmembrane alpha-helices. Nat. Struct. Biol. 1, 157–163. 10.1038/nsb0394-157 7656033

[B49] Lenferink A. E. Pinkas-Kramarski R. van de Poll M. L. van Vugt M. J. Klapper L. N. Tzahar E.. (1998). Differential endocytic routing of homo- and hetero-dimeric ErbB tyrosine kinases confers signaling superiority to receptor heterodimers. EMBO J. 17, 3385–3397. 10.1093/emboj/17.12.3385 9628875 PMC1170676

[B50] Levitzki A. Mishani E. (2006). Tyrphostins and other tyrosine kinase inhibitors. Annu. Rev. Biochem. 75, 93–109. 10.1146/annurev.biochem.75.103004.142657 16756486

[B51] Littlefield P. Liu L. Mysore V. Shan Y. Shaw D. E. Jura N. (2014). Structural analysis of the EGFR/HER3 heterodimer reveals the molecular basis for activating HER3 mutations. Sci. Signal. 7:ra114. 10.1126/scisignal.2005786 25468994 PMC4492339

[B52] Liu J. Hu J. Cheng L. Ren W. Yang M. Liu B.. (2016). Biomarkers predicting resistance to epidermal growth factor receptor-targeted therapy in metastatic colorectal cancer with wild-type KRAS. Onco. Targets. Ther. 9, 557–565. 10.2147/OTT.S86966 26869800 PMC4734822

[B53] Lowenstein E. J. Daly R. J. Batzer A. G. Li W. Margolis B. Lammers R.. (1992). The SH2 and SH3 domain-containing protein GRB2 links receptor tyrosine kinases to ras signaling. Cell 70, 431–442. 10.1016/0092-8674(92)90167-B 1322798

[B54] Luque-Cabal M. García-Teijido P. Fernández-Pérez Y. Sánchez-Lorenzo L. Palacio-Vazquez I. (2016). Mechanisms behind the resistance to trastuzumab in HER2-amplified breast cancer and strategies to overcome it. Clin. Med. Insights Oncol. 10(Suppl. 1), 21–30. 10.4137/CMO.S34537 27042153 PMC4811269

[B55] Macdonald-Obermann J. L. Adak S. Landgraf R. Piwnica-Worms D. Pike L. J. (2013). Dynamic analysis of the epidermal growth factor (EGF) receptor-ErbB2-ErbB3 protein network by luciferase fragment complementation imaging. J. Biol. Chem. 288, 30773–30784. 10.1074/jbc.M113.489534 24014028 PMC3798547

[B56] McDonagh C. F. Huhalov A. Harms B. D. Adams S. Paragas V. Oyama S.. (2012). Antitumor activity of a novel bispecific antibody that targets the ErbB2/ErbB3 oncogenic unit and inhibits heregulin-induced activation of ErbB3. Mol. Cancer Ther. 11, 582–593. 10.1158/1535-7163.MCT-11-0820 22248472

[B57] Mendrola J. M. Berger M. B. King M. C. Lemmon M. A. (2002). The single transmembrane domains of ErbB receptors self-associate in cell membranes. J. Biol. Chem. 277, 4704–4712. 10.1074/jbc.M108681200 11741943

[B58] Meyer A. S. Miller M. A. Gertler F. B. Lauffenburger D. A. (2013). The receptor AXL diversifies EGFR signaling and limits the response to EGFR-targeted inhibitors in triple-negative breast cancer cells. Sci. Signal. 6:ra66. 10.1126/scisignal.2004155 23921085 PMC3947921

[B59] Mueller K. L. Yang Z. Q. Haddad R. Ethier S. P. Boerner J. L. (2010). EGFR/Met association regulates EGFR TKI resistance in breast cancer. J. Mol. Signal. 5:8. 10.1186/1750-2187-5-8 20624308 PMC2911419

[B60] Nahta R. Yuan L. X. Zhang B. Kobayashi R. Esteva F. J. (2005). Insulin-like growth factor-I receptor/human epidermal growth factor receptor 2 heterodimerization contributes to trastuzumab resistance of breast cancer cells. Cancer Res. 65, 11118–11128. 10.1158/0008-5472.CAN-04-3841 16322262

[B61] Nguyen K. S. Kobayashi S. Costa D. B. (2009). Acquired resistance to epidermal growth factor receptor tyrosine kinase inhibitors in non-small-cell lung cancers dependent on the epidermal growth factor receptor pathway. Clin. Lung Cancer 10, 281–289. 10.3816/CLC.2009.n.039 19632948 PMC2758558

[B62] Nolting M. Schneider-Merck T. Trepel M. (2014). Lapatinib. Recent Results Cancer Res. 201, 125–143. 10.1007/978-3-642-54490-3_7 24756789

[B63] Normanno N. De Luca A. Bianco C. Strizzi L. Mancino M. Maiello M. R.. (2006). Epidermal growth factor receptor (EGFR) signaling in cancer. Gene 366, 2–16. 10.1016/j.gene.2005.10.018 16377102

[B64] Ogiso H. Ishitani R. Nureki O. Fukai S. Yamanaka M. Kim J. H.. (2002). Crystal structure of the complex of human epidermal growth factor and receptor extracellular domains. Cell 110, 775–787. 10.1016/S0092-8674(02)00963-7 12297050

[B65] Olayioye M. A. Neve R. M. Lane H. A. Hynes N. E. (2000). The ErbB signaling network: receptor heterodimerization in development and cancer. EMBO J. 19, 3159–3167. 10.1093/emboj/19.13.3159 10880430 PMC313958

[B66] Oxnard G. R. Arcila M. E. Chmielecki J. Ladanyi M. Miller V. A. Pao W. (2011). New strategies in overcoming acquired resistance to epidermal growth factor receptor tyrosine kinase inhibitors in lung cancer. Clin. Cancer Res. 17, 5530–5537. 10.1158/1078-0432.CCR-10-2571 21775534 PMC3166976

[B67] Pelicci G. Lanfrancone L. Grignani F. McGlade J. Cavallo F. Forni G.. (1992). A novel transforming protein (SHC) with an SH2 domain is implicated in mitogenic signal transduction. Cell 70, 93–104. 10.1016/0092-8674(92)90536-L 1623525

[B68] Perrone F. Da Riva L. Orsenigo M. Losa M. Jocollè G. Millefanti C.. (2009). PDGFRA, PDGFRB, EGFR, and downstream signaling activation in malignant peripheral nerve sheath tumor. Neuro. Oncol. 11, 725–736. 10.1215/15228517-2009-003 19246520 PMC2802393

[B69] Qiu C. Tarrant M. K. Choi S. H. Sathyamurthy A. Bose R. Banjade S.. (2008). Mechanism of activation and inhibition of the HER4/ErbB4 kinase. Structure 16, 460–467. 10.1016/j.str.2007.12.016 18334220 PMC2858219

[B70] Riese D. J. II Stern D. F. (1998). Specificity within the EGF family/ErbB receptor family signaling network. Bioessays 20, 41–48. 9504046 10.1002/(SICI)1521-1878(199801)20:1<41::AID-BIES7>3.0.CO;2-V

[B71] Riese D. J. II van Raaij T. M. Plowman G. D. Andrews G. C. Stern D. F. (1995). The cellular response to neuregulins is governed by complex interactions of the erbB receptor family. Mol. Cell. Biol. 15, 5770–5776. 10.1128/MCB.15.10.5770 7565730 PMC230829

[B72] Rubin I. Yarden Y. (2001). The basic biology of HER2. Ann. Oncol. 12(Suppl. 1), S3–S8. 10.1093/annonc/12.suppl_1.S3 11521719

[B73] Sacco A. G. Cohen E. E. (2015). Current Treatment Options for Recurrent or Metastatic Head and Neck Squamous Cell Carcinoma. J. Clin. Oncol. 33, 3305–3313. 10.1200/JCO.2015.62.0963 26351341

[B74] Sacco A. G. Worden F. P. (2016). Molecularly targeted therapy for the treatment of head and neck cancer: a review of the ErbB family inhibitors. Onco. Targets. Ther. 9, 1927–1943. 10.2147/OTT.S93720 27110122 PMC4831599

[B75] Safavi S. Järnum S. Vannas C. Udhane S. Jonasson E. Tomic T. T.. (2016). HSP90 inhibition blocks ERBB3 and RET phosphorylation in myxoid/round cell liposarcoma and causes massive cell death *in vitro* and *in vivo*. Oncotarget 7, 433–445. 10.18632/oncotarget.6336 26595521 PMC4808009

[B76] Saito Y. Haendeler J. Hojo Y. Yamamoto K. Berk B. C. (2001). Receptor heterodimerization: essential mechanism for platelet-derived growth factor-induced epidermal growth factor receptor transactivation. Mol. Cell. Biol. 21, 6387–6394. 10.1128/MCB.21.19.6387-6394.2001 11533228 PMC99786

[B77] Salomon D. S. Brandt R. Ciardiello F. Normanno N. (1995). Epidermal growth factor-related peptides and their receptors in human malignancies. Crit. Rev. Oncol. Hematol. 19, 183–232. 10.1016/1040-8428(94)00144-I 7612182

[B78] Sawma P. Roth L. Blanchard C. Bagnard D. Crémel G. Bouveret E.. (2014). Evidence for new homotypic and heterotypic interactions between transmembrane helices of proteins involved in receptor tyrosine kinase and neuropilin signaling. J. Mol. Biol. 426, 4099–4111. 10.1016/j.jmb.2014.10.007 25315821

[B79] Schlessinger J. (2000). Cell signaling by receptor tyrosine kinases. Cell 103, 211–225. 10.1016/S0092-8674(00)00114-8 11057895

[B80] Schlessinger J. Plotnikov A. N. Ibrahimi O. A. Eliseenkova A. V. Yeh B. K. Yayon A.. (2000). Crystal structure of a ternary FGF-FGFR-heparin complex reveals a dual role for heparin in FGFR binding and dimerization. Mol. Cell 6, 743–750. 10.1016/S1097-2765(00)00073-3 11030354

[B81] Schulze W. X. Deng L. Mann M. (2005). Phosphotyrosine interactome of the ErbB-receptor kinase family. Mol. Syst. Biol. 1, 2005–2008. 10.1038/msb4100012 16729043 PMC1681463

[B82] Sepp-Lorenzino L. Eberhard I. Ma Z. Cho C. Serve H. Liu F.. (1996). Signal transduction pathways induced by heregulin in MDA-MB-453 breast cancer cells. Oncogene 12, 1679–1687. 8622888

[B83] Sergina N. V. Rausch M. Wang D. Blair J. Hann B. Shokat K. M.. (2007). Escape from HER-family tyrosine kinase inhibitor therapy by the kinase-inactive HER3. Nature 445, 437–441. 10.1038/nature05474 17206155 PMC3025857

[B84] Sharma S. V. Bell D. W. Settleman J. Haber D. A. (2007). Epidermal growth factor receptor mutations in lung cancer. Nat. Rev. Cancer 7, 169–181. 10.1038/nrc2088 17318210

[B85] Simon N. FitzGerald D. (2016). Immunotoxin therapies for the treatment of epidermal growth factor receptor-dependent cancers. Toxins (Basel). 8:137. 10.3390/toxins8050137 27153091 PMC4885052

[B86] Slamon D. J. Clark G. M. Wong S. G. Levin W. J. Ullrich A. McGuire W. L. (1987). Human breast cancer: correlation of relapse and survival with amplification of the HER-2/neu oncogene. Science 235, 177–182. 10.1126/science.3798106 3798106

[B87] Soltoff S. P. Carraway K. L. III Prigent S. A. Gullick W. G. Cantley L. C. (1994). ErbB3 is involved in activation of phosphatidylinositol 3-kinase by epidermal growth factor. Mol. Cell. Biol. 14, 3550–3558. 10.1128/MCB.14.6.3550 7515147 PMC358722

[B88] Sorkin A. Goh L. K. (2009). Endocytosis and intracellular trafficking of ErbBs. Exp. Cell Res. 315, 683–696. 10.1016/j.yexcr.2008.07.029 19278030 PMC2605728

[B89] Stern D. F. (2003). ErbBs in mammary development. Exp. Cell Res. 284, 89–98. 10.1016/S0014-4827(02)00103-9 12648468

[B90] Stern D. F. (2008). ERBB3/HER3 and ERBB2/HER2 duet in mammary development and breast cancer. J. Mammary Gland Biol. Neoplasia 13, 215–223. 10.1007/s10911-008-9083-7 18454306 PMC6590701

[B91] Stern H. M. (2012). Improving treatment of HER2-positive cancers: opportunities and challenges. Sci. Transl. Med. 4:127rv2. 10.1126/scitranslmed.3001539 22461643

[B92] Tagliabue E. Castiglioni F. Ghirelli C. Modugno M. Asnaghi L. Somenzi G.. (2000). Nerve growth factor cooperates with p185(HER2) in activating growth of human breast carcinoma cells. J. Biol. Chem. 275, 5388–5394. 10.1074/jbc.275.8.5388 10681513

[B93] Tanizaki J. Okamoto I. Sakai K. Nakagawa K. (2011). Differential roles of trans-phosphorylated EGFR, HER2, HER3, and RET as heterodimerisation partners of MET in lung cancer with MET amplification. Br. J. Cancer 105, 807–813. 10.1038/bjc.2011.322 21847121 PMC3171021

[B94] Therkildsen C. Bergmann T. K. Henrichsen-Schnack T. Ladelund S. Nilbert M. (2014). The predictive value of KRAS, NRAS, BRAF, PIK3CA and PTEN for anti-EGFR treatment in metastatic colorectal cancer: a systematic review and meta-analysis. Acta Oncol. 53, 852–864. 10.3109/0284186X.2014.895036 24666267

[B95] Thery J. C. Spano J. P. Azria D. Raymond E. Penault Llorca F. (2014). Resistance to human epidermal growth factor receptor type 2-targeted therapies. Eur. J. Cancer 50, 892–901. 10.1016/j.ejca.2014.01.003 24462377

[B96] Wallasch C. Weiss F. U. Niederfellner G. Jallal B. Issing W. Ullrich A. (1995). Heregulin-dependent regulation of HER2/neu oncogenic signaling by heterodimerization with HER3. EMBO J. 14, 4267–4275. 7556068 10.1002/j.1460-2075.1995.tb00101.x PMC394510

[B97] Wang Z. Zhang L. Yeung T. K. Chen X. (1999). Endocytosis deficiency of epidermal growth factor (EGF) receptor-ErbB2 heterodimers in response to EGF stimulation. Mol. Biol. Cell 10, 1621–1636. 10.1091/mbc.10.5.1621 10233167 PMC30486

[B98] Wehrman T. He X. Raab B. Dukipatti A. Blau H. Garcia K. C. (2007). Structural and mechanistic insights into nerve growth factor interactions with the TrkA and p75 receptors. Neuron 53, 25–38. 10.1016/j.neuron.2006.09.034 17196528

[B99] Westphal M. Meima L. Szonyi E. Lofgren J. Meissner H. Hamel W.. (1997). Heregulins and the ErbB-2/3/4 receptors in gliomas. J. Neurooncol. 35, 335–346. 10.1023/A:1005837122181 9440030

[B100] Worthylake R. Opresko L. K. Wiley H. S. (1999). ErbB-2 amplification inhibits down-regulation and induces constitutive activation of both ErbB-2 and epidermal growth factor receptors. J. Biol. Chem. 274, 8865–8874. 10.1074/jbc.274.13.8865 10085130

[B101] Yarden Y. Sliwkowski M. X. (2001). Untangling the ErbB signalling network. Nat. Rev. Mol. Cell Biol. 2, 127–137. 10.1038/35052073 11252954

[B102] Zhang X. Gureasko J. Shen K. Cole P. A. Kuriyan J. (2006). An allosteric mechanism for activation of the kinase domain of epidermal growth factor receptor. Cell 125, 1137–1149. 10.1016/j.cell.2006.05.013 16777603

[B103] Zheng Y. Zhang C. Croucher D. R. Soliman M. A. St-Denis N. Pasculescu A.. (2013). Temporal regulation of EGF signalling networks by the scaffold protein Shc1. Nature 499, 166–171. 10.1038/nature12308 23846654 PMC4931914

[B104] Zhuang G. Brantley-Sieders D. M. Vaught D. Yu J. Xie L. Wells S.. (2010). Elevation of receptor tyrosine kinase EphA2 mediates resistance to trastuzumab therapy. Cancer Res. 70, 299–308. 10.1158/0008-5472.CAN-09-1845 20028874 PMC3859619

